# Competitive suppression of dengue virus replication occurs in chikungunya and dengue co-infected Mexican infants

**DOI:** 10.1186/s13071-018-2942-1

**Published:** 2018-07-03

**Authors:** Mussaret B Zaidi, Julio Garcia-Cordero, Ricardo Rivero-Gomez, Josselin Corzo-Gomez, María Elena González y Almeida, Raúl Bonilla-Moreno, José Bustos-Arriaga, Nicolás Villegas-Sepulveda, Leopoldo Flores-Romo, Leticia Cedillo-Barron

**Affiliations:** 1 Department of Molecular Biomedicine, CINVESTAV-IPN, México City, México; 2 Infectious Diseases Research Unit, Hospital General O’Horan, Merida, Mexico; 3 Department of Cell Biology, CINVESTAV-IPN, Mexico City, Mexico; 4 Department of Epidemiology and Biostatistics, Michigan State University, Lansing, USA; 5 Molecular Biology and Arbovirus Immunology UBIMED FES Iztacala, Mexican National Autonomous University, Edo de Mexico, Mexico

**Keywords:** Dengue virus, Chikungunya virus, Co-infection, Disease severity, Maternal antibodies, Seroconversion, Viral competition

## Abstract

**Background:**

Co-circulation of dengue virus (DENV) and chikungunya virus (CHIKV) is increasing worldwide but information on the viral dynamics and immune response to DENV-CHIKV co-infection, particularly in young infants, is scant.

**Methods:**

Blood samples were collected from 24 patients, aged 2 months to 82 years, during a CHIKV outbreak in Mexico. DENV and CHIKV were identified by RT-PCR; ELISA was used to detect IgM and IgG antibodies. CHIKV PCR products were cloned, sequenced and subjected to BLAST analysis. To address serological findings, HMEC-1 and Vero cells were inoculated with DENV-1, DENV-2 and CHIKV alone and in combination (DENV-2-CHIKV and DENV-1-CHIKV); viral titers were measured at 24, 48 and 72 h.

**Results:**

Nine patients (38%) presented co-infection, of who eight were children. None of the patients presented severe illness. Sequence analysis showed that the circulating CHIKV virus belonged to the Asian lineage. Seroconversion to both viruses was only observed in the four patients five years or older, while the five infants under two years of age only seroconverted to CHIKV. Viral titers in the CHIKV mono-infected cells were greater than in the DENV-1 and DENV-2 mono-infected cells. Furthermore, we observed significantly increased CHIKV progeny and reduction of DENV progeny in the co-infected cells.

**Conclusions:**

In our population, DENV-CHIKV co-infection was not associated with increased clinical severity. Our *in vitro* assay findings strongly suggest that the lack of DENV IgG conversion in the co-infected infants is due to suppression of DENV replication by the Asian lineage CHIKV. The presence of maternal antibody and immature immune responses in the young infants may also play a role.

**Electronic supplementary material:**

The online version of this article (10.1186/s13071-018-2942-1) contains supplementary material, which is available to authorized users.

## Background

Over the last few decades, dengue virus (DENV) has spread throughout most tropical and subtropical areas of the world where it has become endemic [1, 2]. Since dengue was reintroduced into the Americas in the 1980s, the region has witnessed numerous outbreaks, with a sharp increase in the number of circulating serotypes and severe cases. Concomitantly, chikungunya virus (CHIKV) has disseminated worldwide since 2004, causing massive outbreaks in Africa, Asia and Latin America. [3]. CHIKV first arrived on the American continent in 2013 [4]; the first autochthonous case from Mexico was reported from the state of Chiapas in October 2014 [5].

DENV and CHIKV are RNA viruses transmitted to humans through the bite of *Aedes* mosquitoes. The broad geographical distribution of the *Aedes aegypti* and *Aedes albopictus* vectors has allowed for the widespread transmission of CHIKV in DENV endemic areas [2]. Vector competence studies have shown that these mosquito species are able to sustain concomitant transmission of both viruses. As a result, human DENV-CHIKV infections may occur through the bite of a co-infected mosquito (co-infections) or sequential bites of mono-infected mosquitoes (superinfection) [6]. A recent study showed that infection, transmission rates and dissemination rates were only mildly affected by double or triple mosquito infection with DENV-2, CHIKV or Zika virus [7].

Both sporadic and outbreak-associated cases of virologically confirmed co-infections have been reported worldwide [6]. The prevalence of DENV-CHIKV co-infection can be remarkably high, particularly during outbreaks. During an Indian epidemic in 2013, for example, up to 83% of DENV infected patients were co-infected with CHIKV [8]. Co-infections have also been detected in Mexico; in all patients, CHIKV isolates belonged to the Asian lineage and were closely related to other isolates from the Western Hemisphere [9, 10].

Several reports have documented a more severe clinical outcome for DENV-CHIKV co-infected patients when compared to mono-infected patients. Notably, all these studies involve the East/Central/South African (ECSA) CHIKV genotype [6].

DENV is highly endemic in southern Mexico, where it has caused major outbreaks since the 1980s [11]. During 2015 alone, the Yucatan State Health Department reported 1669 confirmed cases of Chikungunya fever. During the same year, DENV serotypes 1, 2 and 4 widely co-circulated throughout the state [12]. Between August and October 2015, less than a year after the arrival of CHIKV in Mexico, our hospital witnessed a sudden and massive influx of patients with acute febrile illness and severe joint pain. A significant proportion of young infants required hospitalization.

Although co-infections and viral-viral interactions have been widely described in nature, few reports address the role of viral co-infections on the course of human illness [13]. The dynamics of viral co-infections are complex: they may lead to direct interactions among the infecting viruses, alteration of host susceptibility and cellular translation, and modification of the host immune response, among others. The timing of each infection is an important issue to consider, specifically whether the host is infected by co-infection or superinfection. In this study we show that co-infection with CHIKV and DENV impairs host responses, thus inducing a positive antibody response against CHIKV virus but not against DENV.

Viral interference and genetic re-assortments are the most common form of interactions among arboviral co-infections [14]. Studies on *Flavivirus*/*Alphavirus* co-infections have yielded conflicting results; two studies have demonstrated interference of CHIKV by DENV in mosquitoes and mosquito cell lines [7, 15]. In contrast, Muturi et al. [14] found that Sindbis virus (an alphavirus) suppressed DENV-4 mosquito cell lines.

Information on viral interactions and the immune response in the human co-infected host is scant [14]. Previous studies show that each arbovirus possesses its own signature, starting at very early steps with type I IFN induction by both DENV and CHIKV, which restricts the propagation of both viruses and of subsequent viral infections. The course of the illness and the dominance of one virus over the other(s) is influenced by viral factors (sequence of infection, replication status and capacity of the viral strains, and further mutations in the viral genome) as well as by host factors [16].

During the outbreak, we were interested in determining whether DENV-CHIKV co-infection was occurring in our patients, and its potential impact on their severity of illness. We unexpectedly found that co-infection in young infants resulted in positive IgG seroconversion against CHIKV but not against DENV. We postulated that viral interference could plausibly explain these results and performed *in vitro* assays to support our assumption. This paper presents the first detailed description of DENV-CHIKV co-infections in children from Yucatan, Mexico, and show that CHIKV-DENV co-infection impairs host responses, which induce memory antibody responses to CHIKV alone.

## Methods

### Setting and sample collection

The patients included in this study were enrolled at the Hospital General O’Horan Emergency Department during a two-week period in September 2015, at the height of the CHIKV outbreak in Yucatan. Patients presented fever greater than 38 °C and two or more of the following symptoms: rash, arthralgia, myalgia and headache. After obtaining written informed consent, clinical data was registered on a standardized questionnaire and blood samples were collected during acute illness on admission or the first medical visit. Whole blood was collected in Trizol tubes (0.5 ml) and serum clot activator tubes (1–8 ml depending on age) on Day 1 to 7, hereby referred to as the acute sample. Hospitalized patients were followed prospectively until discharge. Patients returned for a follow-up visit 4–6 weeks after onset of illness for a full physical examination and collection of convalescent sera (hereby referred to as convalescent samples). Trizol tubes were stored at -70 °C until RNA extraction. Serum activator tubes were centrifuged for 15 min at 2000× *g*; serum samples were aliquoted and stored at -20 °C until use.

### Viruses and virus titrations

The following viral isolates were used for the cell assays: A CHIKV strain from a clinically ill patient from the state of Yucatan in 2015 (GenBank: MF407264); a DENV-2 strain with high nucleotide sequence homology to the New Guinea strain [17], obtained from a patient on the Mexican east coast in 1997; and a DENV-1 strain obtained from a clinically ill patient from the state of Yucatan in 2015. Viral stocks were collected from cell culture supernatants and stored at -70 °C.

Virus titers were determined by plaque assays. Briefly, 24-well plates were seeded with Vero cells in duplicate, and when they reached 80–90% confluence; serial 10-fold dilutions were made of supernatants collected from the different experimental conditions (CHIKV, DENV or CHIKV-DENV co-infection). Infected Vero cells were then completely overlaid with DMEM containing methylcellulose and maintained for 4 days at 37 °C. The overlays were removed with gentle washes and fixed with methanol at 80% and the monolayers were blocked with 5% PBS-milk. After washing, one plate was incubated with mouse IgG antibody 4G2 at 1:2000 (anti DENV E protein) and the second plate was incubated with mouse IgG at 1:500 (anti E2 CHIKV). Subsequently, anti-mouse IgG (H+L) was added to the assay at a 1:2000 dilution with horseradish peroxidase (HRP) and incubated for 1 h. Plates were washed and True Blue was added to reveal the plaques. Stock viruses were prepared by infecting a C6/36 cell monolayer in 75 cm^2^ tissue culture flasks at 75–85% confluence.

### Flow cytometry

Infected Vero cells (0.25 MOI) were analyzed at 48 h post-infection. Cells were detached with trypsin solution and treated with trypsin-neutralizing solution (Lonza Group Ltd., Basel, Switzerland). Once in suspension, cells were treated with fixing/permeabilising solution (Becton Dickinson, New Jersey, USA) at 4 °C for 1 h with gentle agitation, washed twice with PBS/0.5% albumin, and treated for 30 min with an unrelated immunoglobulin solution (10% goat serum in PBS). Cells were then incubated with rat anti-NS5 monoclonal antibody for DENV [18] and with a mouse polyclonal anti E2 for CHIKV. After 30 min incubation, the cells were treated with the corresponding secondary antibodies: anti-mouse IgG H+L CY3 and anti-rat-FITC IgG H+L. Data were collected using the FACS Dako Cyan ADP analyzer (Beckman Coulter, Pasadena, California, USA) and data analysis was performed using FlowJo (Tree Star Inc., Ashland, Oregon, USA).

### Detection of DENV and CHIKV antibodies in serum samples

The detection of anti-DENV and anti-CHIKV IgM and IgG was performed with commercial ELISA kits (DRG Diagnostics, Springfield, USA); all assays were run in duplicate. The optical density (OD) was measured at 450 nm and the units of antibody concentration and cut-off values were calculated as described by the manufacturer. A positive IgG seroconversion was defined as the change from a negative IgG serological value in the acute sample to a positive IgG value in the convalescent serum sample.

### Isolation of DENV and CHIKV and amplification of PCR

Trizol was used for extraction of viral RNA from the acute samples (Invitrogen, Carlsbad, CA, USA) according to the manufacturer’s instructions. Retrotranscription of cDNA was performed with a Superscript III reverse transcriptase kit (Invitrogen). DENV PCR was performed according to the methods described by Lanciotti et al. [19] (D1: 5'-TCA ATA TGC TGA AAC GCG CGA GAA ACC G-3' from 134 to 161; and D2: 5'-TTG CAC CAA CAG TCA ATG TCT TCA GGT TC-3' from 616 to 644), in which the desired product was a 511 bp amplicon. For the CHIKV PCR we used primers for the E2 gene sequence (E2F: 5'-GCG CGT GGG TGC GT-3'; and E2R: 5'-TTT CGG CTA AAT GCT CG-3'). PCR amplification was performed in an Applied Biosystems 2720 Thermal cycler (Thermo Fisher Scientific, Waltham, USA) using the following conditions: 94 °C for 5 min, followed by 25 cycles of 94 °C for 30 s, 52 °C for 30 s, 72 °C for 30 s and 10 cycles of 94 °C for 30 s, 56 °C for 30 s, and 72 °C for 30 s, with a final extension of 72 °C for 7 min. An amplicon of 1032 bp indicated the presence of the CHIKV E2 sequence. We used plasmids that contained previously cloned and sequenced fragments of E2 CHIKV protein and cDNA from DENV-2 infected cells to amplify C-prM protein as positive controls.

DENV-CHIKV co-infection was defined as positive PCR gene amplification for both viruses, a positive PCR test for a single virus and positive IgG seroconversion to the other virus or negative PCR results but positive IgG seroconversion results for both viruses.

### CHIKV sequencing and phylogenetic analysis

The CHIKV PCR fragment was cloned using the PCR-XL TOPO vector according to the manufacturer’s instructions (Invitrogen). The Topo vector containing a 1032 bp fragment of CHIKV was sequenced in the forward and reverse directions, and sequences were submitted to the GenBank database. The viral sequences were aligned, analysed and subjected to homology search by BLAST analysis. Analysis of nucleotide sequences and deduced amino acid sequences were performed by using EXPASY Tools [19] and Clustal W v.2.0 (https://www.expasy.org/tools/). Phylogenetic trees were constructed by using the neighbour-joining algorithm as implemented in PHYLIP (https://www.ebi.ac.uk/Tools/msa/clustalw2/) [20].

### DENV-CHIKV co-infection assays

Because the young co-infected infants underwent IgG seroconversion to CHIKV but not to DENV, we performed co-infection experiments in both HMEC-1 and Vero cells to assess whether this could be due to DENV suppression by CHIKV. Co-infection assays were performed *in vitro* by using Vero and HMEC-1 cells infected with CHIKV, DENV-1 and DENV-2 strains obtained from Mexican subjects during the current study (DENV-1 and CHIKV) or previous studies conducted by our team (DENV2). DENV-1 was selected because it was the predominant serotype in Yucatan during 2015 and DENV-2 was selected, as it was the most widespread serotype in Mexico during recent years. Vero cells (6 × 10^4^), highly permissive for both DENV and CHIKV, were seeded on glass coverslips (Bellco, New Jersey, USA) in 24-well plates and incubated overnight. The monolayers of Vero cells were infected at MOI of 0.25 for DENV-1/2 and CHIKV and the monolayers of HMEC-1 cells were infected at MOI of 2.5 for DENV-1/-2 and CHIKV; both were placed in a shaking incubator for 2 h at 37 °C. Upon removal, cells were washed with PBS and supplemented with complete medium. Cells were subsequently incubated at 12, 24, and 48 h at 37 °C, stained by immunofluorescence, and viral titers were measured from the supernatants of Vero cells at each time point [21]. Mock-infected and uninfected cells served as negative controls. Briefly, cells were fixed with 4% paraformaldehyde (Sigma-Aldrich, St. Louis, MO, USA) in PBS for 20 min at room temperature, permeabilized with a solution of PBS supplemented with 0.1% Triton-X100 and blocked with 10% normal goat serum. The cell monolayer was incubated for 60 min with primary antibodies as follows: mAb 4G2 for detection of DENV-1, anti-NS5 for DENV-2 or anti-E2 protein for CHIKV [18]. After washing, the following fluorochrome-conjugated secondary antibodies were added: mouse IgG H+L for DENV-1 (Invitrogen, Carlsbad, CA, USA) or rat IgG H+L for DENV-2 and CHIKV (Invitrogen, Carlsbad, CA, USA). Irrelevant isotype antibodies were used as a negative control. Nuclei were labelled with DAPI (1 μg/ml) in PBS for 10 min, and the slides were mounted with VECTASHIELD (Vector Labs, Burlingame, CA, USA). The images were captured with a confocal microscope (Leica SP2, Barcelona, Spain).

## Results

### Clinical characteristics, RT-PCR and serological results

We included 24 patients aged 2 months to 82 years. More than half of them (63%) were children, six of whom (25%) were less than one year of age. Among the 24 patients, 14 (58%) had positive PCR results. Three patients were positive for DENV only, five for CHIKV only and six for both viruses (Table 1). Convalescent serum samples were obtained from 21 (88%) of the 24 patients at 4 to 6 weeks post-infection. Of the three DENV-PCR-positive patients, one (#150) did not return for convalescent sampling. The two remaining patients (#151 and #152) presented DENV and CHIKV specific IgM in their acute samples and seroconversion of DENV and CHIKV-specific IgG antibodies, and were classified as co-infected. Of the five CHIKV-PCR-positive patients, one patient (#129) also had positive DENV IgM in the acute sample but was lost to follow-up. Another patient (#130) presented positive seroconversion to DENV and CHIKV and was classified as co-infected. The three remaining patients (#139, #157 and #166) presented IgG seroconversion to CHIKV alone (Table 1). The combined PCR and serology results yielded nine DENV-CHIKV co-infections (38%), two DENV only infections (8%) and four CHIKV only infections (17%). Of the six children who were DENV and CHIKV-PCR-positive, five, who were infants less than two years of age (#147, #155, #170, #178, and #180), presented positive seroconversion of CHIKV-specific IgG antibodies, but no seroconversion of DENV-specific IgG antibodies. Seroconversion to both viruses was only observed in three children aged five years or more (#130, #137, #152) and a young adult (#151).

**Table 1:** PCR and ELISA results for chikungunya and dengue infection in patients with acute febrile disease, Yucatan, Mexico

Patient number	Age	Days of onset	H/A	CHIKV PCR	DENV PCR	DENV IgM acute	DENV IgG acute	DENV IgM conv	DENV IgG conv	CHIKV IgM acute	CHIKV IgG acute	CHIKV IgM conv	CHIKV IgG conv	Final Diagnosis
125	23 y	7	H	-	-	-	+	-	+	-	-	-	-	Negative
129	27 y	5	A	+	-	+	+	na	na	-	-	na	na	Chikungunya
130	5 y	1	H	+	-	-	-	-	+	-	-	-	+	Co-infection
132	2 m	1	H	-	-	-	+	-	-	-	-	-	-	Negative
137	8 y	2	A	+	+	+	+	-	+	-	-	+	+	Co-infection
139	27 y	4	A	+	-	-	+	-	+	-	-	+	+	Chikungunya
141	5 m	3	A	-	-	-	+	-	+	-	-	-	-	Negative
146	33 y	2	A	-	-	-	+	-	+	-	-	-	-	Negative
147	4 m	2	H	+	+	-	-	-	-	-	-	-	+	Co-infection
149	3 y	2	A	-	-	-	-	na	na	-	-	na	na	Negative
150	5 y	2	A	-	+	-	-	na	na	-	-	na	na	Dengue
151	20 y	5	A	-	+	+	+	-	+	+	-	-	+	Co-infection
152	7 y	5	H	-	+	+	-	-	+	+	-	+	+	Co-infection
154	15 y	3	A	-	-	+	-	-	+	-	-	-	-	Dengue
155	21 m	2	A	+	+	+	-	-	-	-	-	-	+	Co-infection
157	12 y	3	A	+	-	-	-	-	-	-	-	-	+	Chikungunya
158	82 y	1	A	-	-	-	+	-	+	-	-	-	-	Negative
160	22 y	2	H	-	-	-	+	-	+	-	-	-	-	Negative
162	30 y	2	A	-	-	-	+	-	+	-	-	+	+	Chikungunya
166	14 y	4	A	+	-	-	+	-	+	-	-	-	+	Chikungunya
170	2 m	1	H	+	+	-	+	+	-	-	-	-	+	Co-infection
174	19 y	3	A	-	-	-	+	-	+	-	-	-	-	Negative
178	2 m	2	H	+	+	-	+	-	-	-	-	-	+	Co-infection
180	3 m	2	H	+	+	-	+	-	-	-	-	-	+	Co-infection

*Abbreviations DENV* dengue virus, *CHIKV* chikungunya virus, *y* years, *m* months, *H* hospitalized, *A* ambulatory, *na* not available

### Clinical presentation of mono-infected and co-infected DENV-CHIKV patients

Of the 15 patients with DENV, CHIKV or DENV-CHIKV co-infection, all had fever and joint or muscle pain, and 93% had rash. The two patients with DENV alone had abdominal pain during acute illness and none of them presented persistent arthralgia at one-month follow-up. Eleven of 13 CHIKV or DENV-CHIKV co-infected patients referred incapacitating arthralgia during the first visit. Two of the four CHIKV-infected patients and two of the nine DENV-CHIKV co-infected patients presented persistent arthralgia at one-month follow-up. None of the patients presented life-threatening illness and we did not observe any distinctive clinical or laboratory features in co-infected patients compared to mono-infected patients. No patients presented thrombocytopenia, and there were no marked differences in leucocytosis, leukopenia or lymphopenia among the patient groups (Table 2).

**Table 2:** Clinical characteristics of patients with dengue and chikungunya monoinfection compared to dengue-chikungunya coinfection

Patient number	Age	Infection status	T (°C)^a^	Presence of rash and distribution	Severe arthralgia/Myalgia^b^	Arthralgia at 30 days	Total leukocytes (×10^3^/ml)	Total lymphocytes (×10^3^/ml)	Total platelets (×10^3^/ml)
130	5 y	Coinfection	38.0	Generalized	Yes	No	11.6 ↑	0.8 ↓	167
137	8 y	Coinfection	39.0	Generalized	Yes	Yes	10.10	0.6 ↓	256
147	4 m	Coinfection	39.1	Generalized including palms and soles	Yes	No	14.9	4.6	245
151	20 y	Coinfection	39.7	Generalized	Yes	No	8.3	2.1	244
152	7 y	Coinfection	38.5	Generalized	No	No	3.7 ↓	1.1 ↓	202
155	21 m	Coinfection	38.3	Generalized	Yes	Yes	10.8	1.4 ↓	258
170	2 m	Coinfection	38.7	Generalized	Yes	No	6.2	1.5 ↓	326
178	2 m	Coinfection	39.0	Generalized	Yes	No	18.6 ↑	2.5 ↓	313
180	3 m	Coinfection	39.2	Generalized	Yes	No	5.1 ↓	3.2 ↓	182
150	5 y	Dengue	41.9	Generalized	No	No	6.8	1.0 ↓	155
154	15 y	Dengue	38.0	Generalized	No	No	4.2	1.1	156
139	27 y	Chikungunya	38.2	No rash	Yes	Yes	3.5 ↓	1.4	153
157	12 y	Chikungunya	38.0	Generalized	Yes	Yes	3.9 ↓	1.4	261
162	30 y	Chikungunya	38.5	Generalized	No	No	6.6	0.6 ↓	163
166	28 d	Chikungunya	38.2	Generalized	Yes	No	10.9	1.4 ↓	277

^a^Temperature at the time of admission

^b^Severe arthralgia/myalgia defined as intense crying upon movement or touching of limbs in young infants (age under 1 year) and incapacitating joint pain in older children and adults

*Abbreviations y* years, *m* months; *D* days; ↓, low count according to age; ↑, high count according to age

### CHIKV sequencing and phylogenetic analysis revealed an Asiatic genotype

**Fig. 1:**
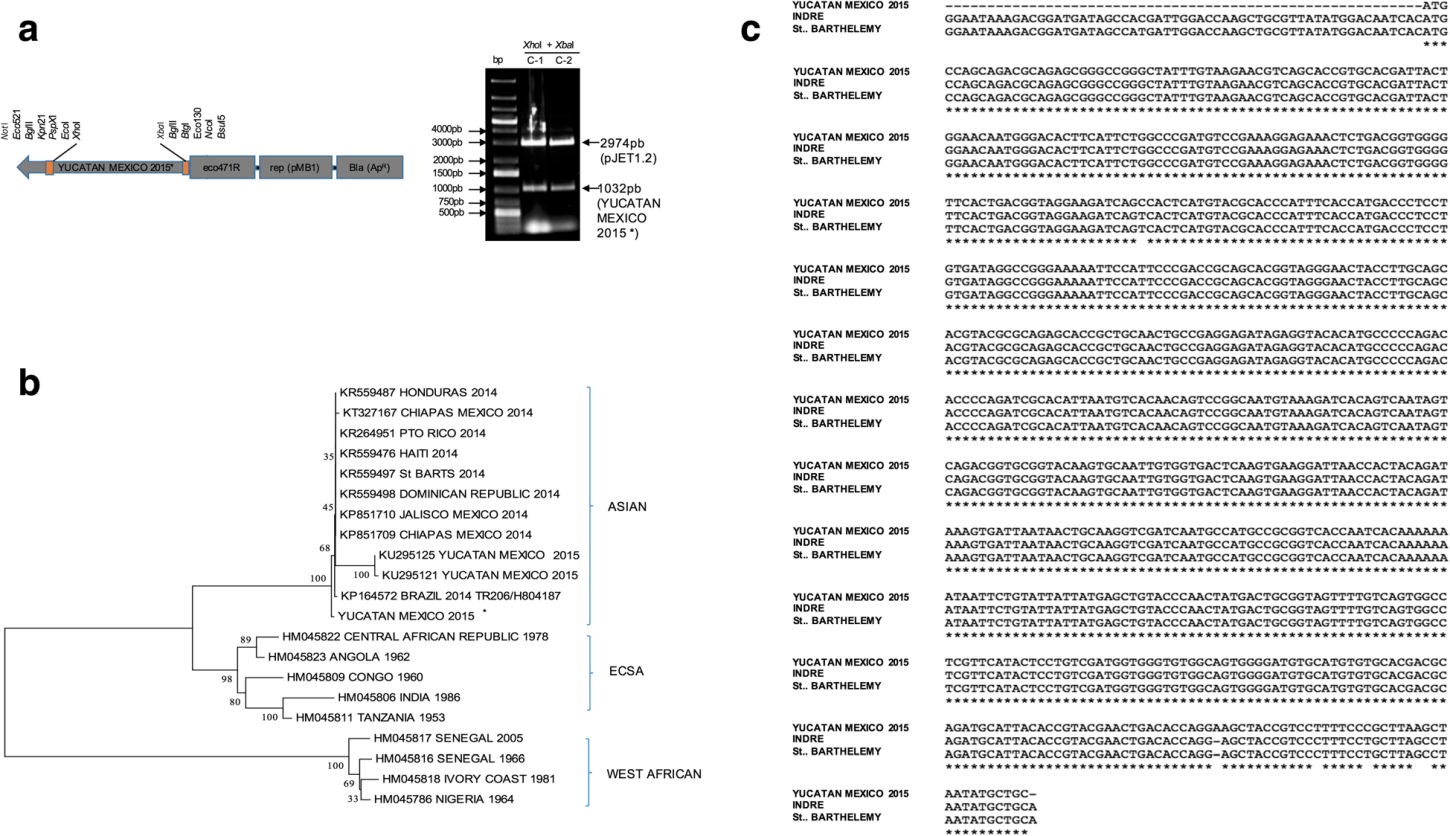
**a** Cassette where the sequence of clinical isolates, were cloned. Agarose gel electrophoresis of RT-PCR products from two positive patients (#137 and #178). Lane 1: molecular weight ladder; Lanes 2 and 3: positive bands corresponding to CHIKV (1032 bp) amplified fragments, (patients #137 and #178), respectively. **b** Phylogenetic tree of CHIKV strains, based on the alignment of 1032 bp of the nucleotide sequence from the structural E-2 protein nucleotide sequence. Each strain is designated by its GenBank accession number followed by country of origin. The Yucatan HOH strain is identical to those previously reported in Yucatan during the same year and has a 100% identity to the strain isolated in Chiapas, Mexico in 2014. **c** Comparative protein sequence of CHIKV cloned

Of the six patients with a positive DENV and CHIKV amplicon, two RT-PCR products were cloned in the TOPO vector (Fig. 1a**)**. We chose three colonies from each patient for sequencing analysis. We then performed a phylogenetic analysis of the CHIKV E2 region comparing our sequences to others available in the GenBank database. The nucleotide sequences of our two isolates possessed 100% identity to each other. Furthermore, the nucleotide sequence of one isolate (GenBank: MF407264) was aligned with a CHIKV sequences previously reported for an isolate from Chiapas, Mexico (100%, GenBank: KP851709.1). The phylogenetic tree showed that our isolates belonged to the Asian genotype (Fig. 1b, c).

### DENV-CHIKV co-infection assays

To quantitatively measure viral titters in Vero and HMEC-1 cells, and considering that the viral antigens are proportional to virus replication, a flow cytometry analysis was performed for co-infected Vero cells at 48 h. Both E2 and NS5 proteins were detected at 48 h post-infection.

CHIKV mono-infected cells yielded 40.9% positive cells (detection of E2 CHIKV protein), and 25.6 % positive cells (detection of NS5 protein) was observed in DENV-2 mono-infected cells. In contrast, when Vero cells were co-infected with DENV-2-CHIKV we observed that the percent of detectable DENV-2 infected cells (NS5 fluorescent cells) dropped to 2.3% while 34.0% of cells were positive for CHIKV (Additional file 1: Figure S1).

**Fig. 2:**
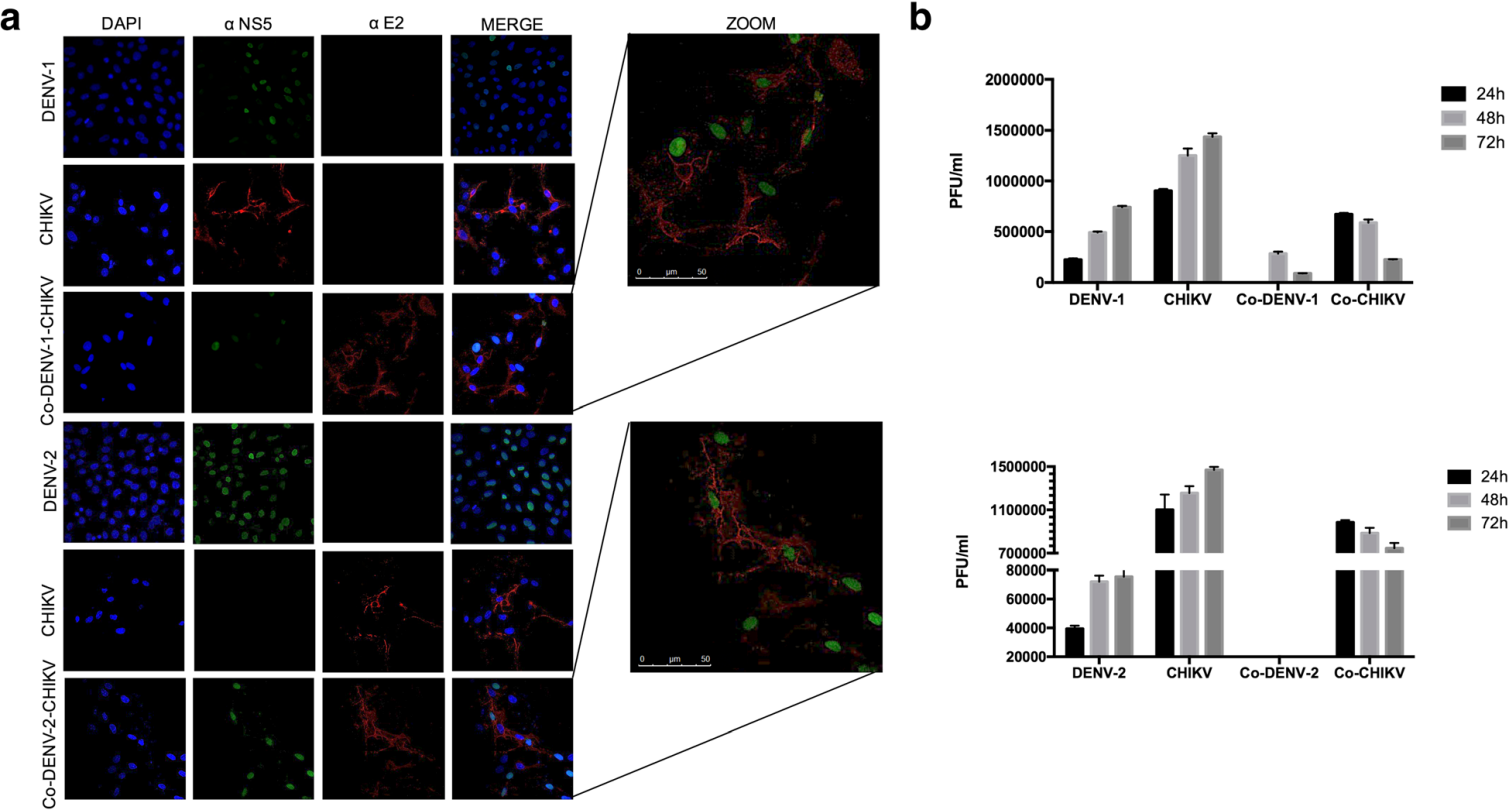
CHIKV suppresses DENV replication in cell culture assays. **a** Confluent monolayers of Vero cells were infected in triplicate, at 0.25 MOIs with a single virus (CHIKV, DENV1) upper panel or DENV-2 bottom panel or co-infected with CHIKV-DENV-1 or CHIKV-DENV-2. **b** Viral titers for DENV-1 or CHIKV mono-infection assays, DENV-1 titers for co-infected cells (Co-DENV-1) and CHIKV titers for co-infected cells (Co-CHIKV) upper panel. Viral titers for DENV-2 and CHIKV mono-infection assays, DENV-2 titers for co-infected cells (Co-DENV) and CHIKV titers for co-infected cells (Co-CHIKV). Viral titers were measured at indicated time points. The data shown represent the averages from at least 3 independent experiments. Standard deviations of the means are shown. Statistical significance was determined with Student’s t-tests

**Fig. 3:**
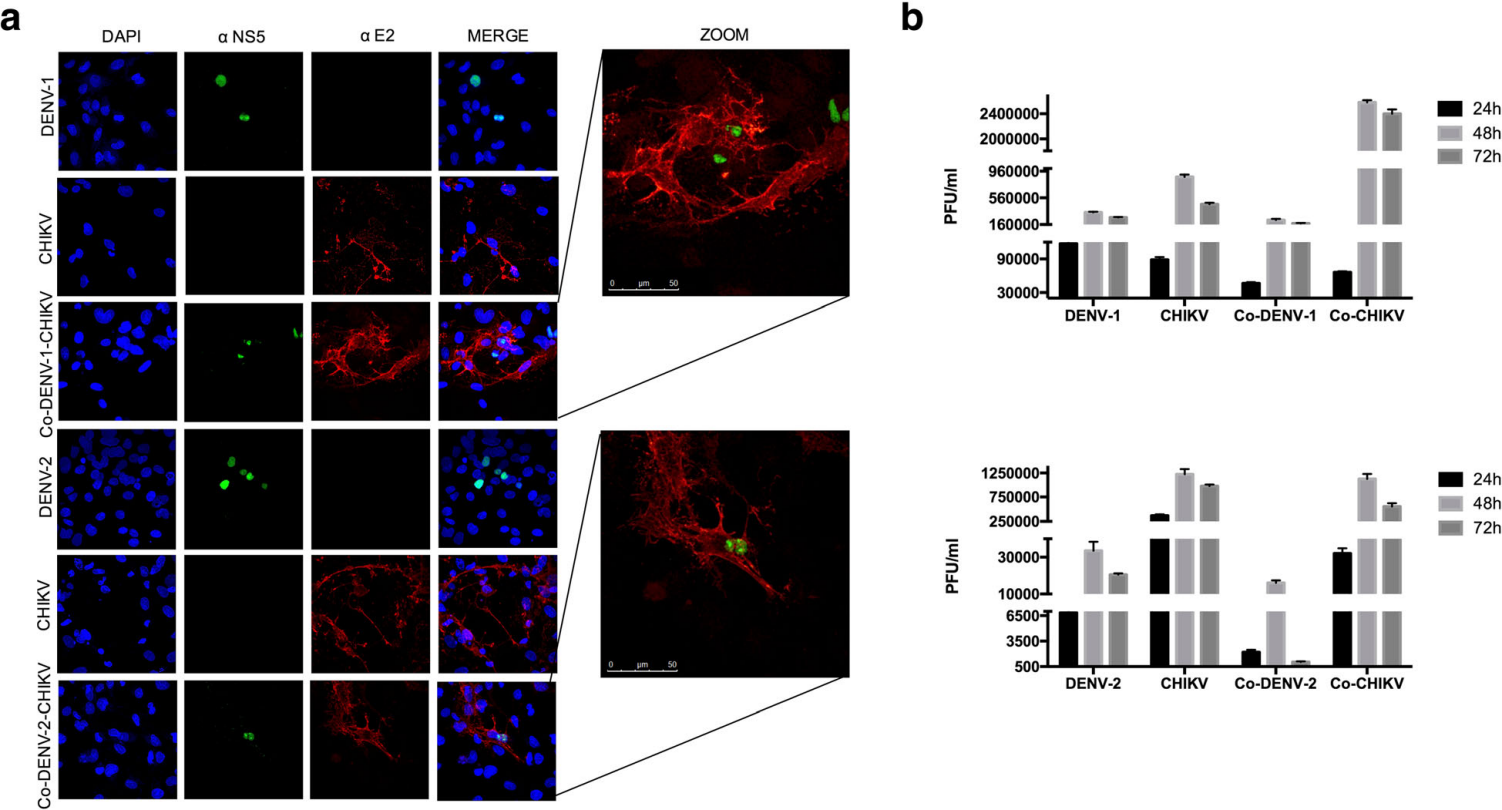
CHIKV suppresses DENV replication in cell culture assays. **a** Confluent monolayers of HMEC-1 cells were infected in triplicate, with a single virus at 2.5 MOIs (CHIKV, DENV1 or DENV-2) or co-infected with CHIKV-DENV-1 or CHIKV-DENV-2 Viral titers for DENV-1 and CHIKV mono-infection assays, DENV-1 titers for co-infected cells (Co-DENV-1) and CHIKV titers for co-infected cells (Co-CHIKV). **b** Viral titers for DENV-2 and CHIKV mono-infection assays), DENV-2 titers for co-infected cells (Co-DENV-2) and CHIKV titers for co-infected cells (Co-CHIKV). Viral titers were measured at indicated time points. The data shown represent the averages from at least 3 independent experiments. Standard deviations of the means are shown. Statistical significance was determined with Student’s t-tests

We performed an immunofluorescence to characterize CHIKV and DENV co-infection. When compared to DENV-1 and DENV-2 mono-infected cells at 24, 48 and 72 h, with CHIKV, mono-infected cells presented earlier and more intense cytopathic effects (Fig. 2a). The immunofluorescence results showed non-structural protein NS5 RNA polymerase in the nucleus of both DENV-2 mono-infected and co-infected cells. Likewise, the presence of E2 CHIKV protein was detected in the cytosol of CHIKV mono-infected and co-infected cells. No signal was detected in the mock-infected cells or uninfected cells. (Figs. 2a and 3a).

To further determine that such DENV-CHIKV co-infection was productive, with the release of new infectious particles, plaque assays were performed. Viral titers in the CHIKV mono-infected cells were greater in both HMEC and Vero cells than in the DENV-1 and DENV-2 mono-infected cells. Furthermore, in the co-infected cells we observe a significant reduction of DENV progeny and few changes in numbers of plaques of CHIKV. However, a clear inhibitory effect of CHIKV on DENV-2 and DENV1 was observed in both cell lines Vero and HMEC-1 cells (Figs. 2b and 3b).

## Discussion

To the best of our knowledge, this is the first report on DENV-CHIKV co-infections in young Mexican infants and provides evidence that the viral dynamics and immune response in this age group differs from that of older children and adults. The prevalence of co-infections in our patients (38%) was remarkably high. Given the small number of patients and the short time period studied, it is not possible to determine whether this reflected the true prevalence of co-infection in the population of Yucatan during the 2015 dengue season. Our findings, however, do support evidence from other countries that DENV-CHIKV co-infections occur frequently during outbreaks [8, 22]. Co-infection rates vary widely worldwide, ranging between 0.9–18.2% in Africa [22–24], 8.7–13.7% in India [8, 25, 26], and more recently, 5% in northwestern Mexico [5]. The high prevalence of human co-infection is likely influenced by a variety of factors such as the density of mosquitoes harboring DENV and/or CHIKV and the frequency of co-infection in the mosquito vector, as well as the density of the human population and the prevalence of neutralizing antibodies to circulating DENV serotypes due to previous exposure [27].

Our CHIKV isolates had a 100% nucleotide identity and a 100% amino acid similarity with the first CHIKV isolated in Mexico, which belonged to the Asian genotype [28]. Moreover, our CHIKV strains had a 100% homology to those previously isolated from Yucatan during 2015 [9]. The data concur with previous reports suggesting that a single strain of the CHIKV Asian genotype spread throughout the Americas [29, 30]. The specific CHIKV genotype is of public health importance as certain investigators [31] have suggested that the CHIKV Asian genotype is associated with greater neurovirulence and higher neuron mortality in mice brain cultures.

The preferential IgG seroconversion of young infants to CHIKV but not to DENV is an interesting and unexpected finding that may have several explanations: (i) CHIKV replicates and disseminates faster than DENV; as a consequence, CHIKV may trigger and simultaneously control the innate immune response through different evasion mechanisms before DENV can induce the innate immune response; (ii) the presence of circulating maternal anti-DENV but not anti-CHIKV antibodies in young infants; IgG may resolve the DENV infection in the infants before a proper adaptive immune response can be initiated; and (iii) intrinsic differences in the immune response of young infants compared to older children and adults.

Several studies [32] have shown that in endemic areas with intense DENV circulation, 90 to 100% of newborns have maternal neutralizing antibodies against the virus. At least three of four serotypes have been circulating in Yucatan during the last decade. Thus, specific antibodies against DENV are highly prevalent in the local population. In Asia, the loss of these transplacentally acquired neutralizing antibodies occurs at 9 to 12 months of age [33–35] but in the Americas region this occurs earlier, around 4 months of age [36]. These maternally derived antibodies appear to play a strong protective role against dengue infection in young infants [34, 36, 37]. Our data suggest that the kinetics of maternal antibodies in our infants from Yucatan were similar to those in Brazil, as five of the six (83%) young infants (2–5 months of age) had anti-DENV IgG in their acute samples. Although it is generally assumed that these protective antibodies are of transplacental origin, a recent study in mice [38] showed that breastfeeding represents the main route of maternal IgG transfer to neonates. Plausibly, the IgG antibodies found in the acute samples of our young infants, and those found in other studies, could actually have been transmitted through breastfeeding.

Vaccine studies have shown that antibodies of maternal origin bind to vaccine antigens and prevent infant B cells from accessing immunodominant vaccine epitopes. Furthermore, these maternal antibodies decrease the antigen load in such a manner that plasma cell differentiation is impaired in young infants [39, 40]. It is likely that in three of our very young co-infected infants (#170, #178 and #180) maternal neutralizing antibodies against DENV not only protected the infants from infection, but also prevented them from developing disease, resulting in an insufficient memory to evoke during the convalescent period.

Our data also show, however, that the presence of maternal virus-specific antibodies in the young infants alone cannot explain the lack of DENV-specific IgG seroconversion, as two other co-infected infants, one four months of age (#147) and an older toddler, 21 months of age (#155) lacked IgG in the acute sample. Another possibility is that some of these children were sequentially infected, with CHIKV being the first virus introduced into the host. This would induce a proper host immune response for CHIKV, but would interfere with a subsequent infection by DENV. A possible mechanism would involve the first virus taking over the cellular factors required for viral replication or interfering with the replication of a secondary viral infection. The interference of one virus by another during co-infection could differentially induce an antiviral state mediated by IFNs. Each virus possesses their own signature, and an effective IgG response by the host depends on which virus first infected the host. Additionally, the efficiency of the nuclear translocation of IRF3- and IRF7 type I IFN-dependent antiviral responses which is the main protagonist to control viral replication and dissemination and also the IFN stimulated response elements (ISRG) located in promoter regions of different ISGs, may be differential depending on the virus involved.

Potiwat et al. [15] studied the effect of ECSA CHIKV and DENV-3 co-infection in mosquito cell cultures. They observed that during mixed infections with equal titers of virus or when CHIKV MOI exceeded that of DENV, both viruses were able to replicate and generate progeny. However, when the proportion of DENV exceeded that of CHIKV, replication in the latter was suppressed. The authors concluded that viral load rather than the order of viral infection was the major determinant for the outcome of these two co-infections. Another study [41] using a DENV-2 strain and an Indian Ocean lineage CHIKV strain from La Reunion, showed an inhibitory effect of DENV on CHIKV infection in PBMCs, in addition to changes in type I IFN production [41]. Yet another study [7] sought to determine vector competence with double or triple infection to DENV, CHIKV and Zika viruses. The authors found that when live mosquitoes co-exposures with both CHIKV and DENV-2, the CHIKV progeny was reduced and only small enhancement of DENV replication was observed. However only mild effects in terms of infection, dissemination and transmission rates in mosquito was observed. Due to our study design it was not possible to establish whether the patients had a superinfection or a true co-infection.

In the co-infection assays that we performed for this study, we observed that CHIKV replication in mono- and co-infected cells exceeded that of DENV-1 and DENV-2 by more than 200-fold. In DENV-CHIKV co-infected cells we detected low DENV-1 viral particles in the supernatant but DENV-2 was still capable of low-level replication. This suggests that in natural conditions the circulating Asian genotype has higher replication rates than DENV, which might lead to the interspecific competition of DENV in co-infected mosquitoes and in human hosts. Recently, investigators observed that DENV was rapidly outcompeted by CHIKV in a cell culture from a DENV-CHIKV co-infected patient from Yucatan, and was attributed to the slower replication rate of DENV [9]. The conflicting results of other DENV-CHIKV co-infection studies with our findings might be explained by differences in the specific genotypes and cell lines. Potiwat et al. [15] used DENV-3 and ECSA CHIKV reference strains while Ruiz-Silva et al. [42] used DENV-2 and the Indian Ocean lineage CHIKV. We used circulating DENV-1 and CHIKV strains during the 2015 outbreak. Furthermore, co-infection dynamics in mosquito cell lines (used by Potiwat et al. [15] may differ from those occurring in Vero, HMEC-1 and human cells, a point that warrants further investigation.

Age-dependent differences in the host-immune response may also partially explain some of our findings. There is ample evidence that young infants have decreased expression of cell surface receptors in naïve B cells and poor IgG responses to protein and polysaccharide antigens. Thus, it is plausible that our older co-infected children have a more finely tuned immune system and are capable of detecting both antigens even at very low levels, thus generating an adaptive response with memory IgG antibodies.

While our study results strongly suggest that the Asian lineage CHIKV suppresses DENV replication, it is important to mention its limitations. The number of co-infected patients we studied was small and it was not possible to determine whether the co-infections were simultaneous or sequential, a factor that may be determinant for viral replication. Future research studies should attempt to include a larger number of subjects from different age groups and to quantify the viral load in ill patients by real-time PCR.

## Conclusions

In conclusion, our study suggests that DENV-CHIKV co-infection occurred in Yucatan, Mexico during 2015 and that these co-infections were not associated with increased clinical severity. Among co-infected patients, older children and adults presented specific IgG that demonstrate an immune response to both viruses, while young infants seroconverted to CHIKV alone. Our findings could be explained by a myriad of factors such as the presence of DENV-reacting maternal antibodies and the immune responses in young infants, the viral competence of the circulating DENV and CHIKV strains, and the timing of the viral infections in the host (co-infection *vs* superinfection). These findings have important implications for the development of polyvalent vaccines that include multiple DENV serotypes or multiple arboviruses such as dengue, Zika and chikungunya. Future vaccine research should elucidate whether suppression of viral replication occurs in young children and whether this plays a critical role in effective vaccine response.

## Additional file

Additional file 1: **Figure S1.** Co-infection of DENV-2 and CHIKV in Vero cells, analyzed by flow cytometry: The Vero cells were infected in a 6-well plate with DENV-2 or CHIKV at MOI of 0.25 in both mono-infection and co-infection assays and then evaluated at 48 h. The cells were harvested from each plate, then double stained assay and flow cytometry analysis were performed to confirm cells infected with DENV anti-NS5 (X) and infected cells with CHIKV anti-E2 (Y). (TIFF 917 kb)

## Abbreviations

DENV: Dengue virus
CHIKV: Chikungunya virus
PBMCs: Peripheral blood mononuclear cells
IFN I: Interferon type I
