# Supplementary figures and images for: Competitive suppression of dengue virus replication occurs in chikungunya and dengue co-infected Mexican infants
Source: Parasit Vectors. 2018 Jul 3;11:378. doi: 10.1186/s13071-018-2942-1 (PMC6029041; doi:10.1186/s13071-018-2942-1)

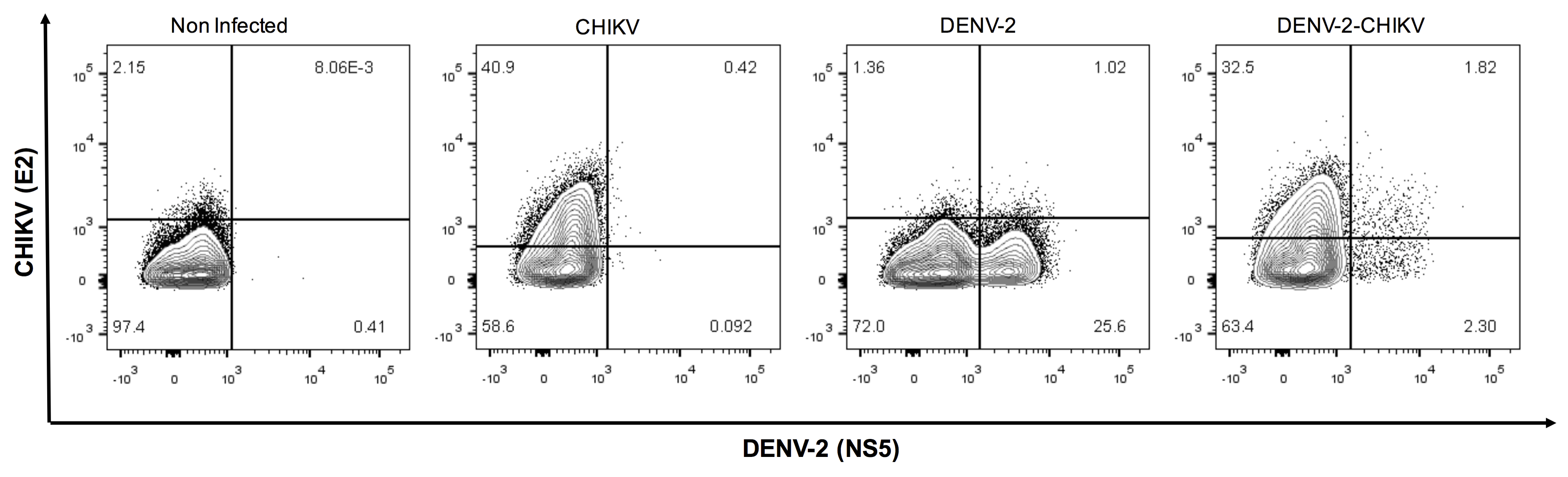

Supplement: Supplementary file 1 — Figure S1. Co-infection of DENV-2 and CHIKV in Vero cells, analyzed by flow cytometry: The Vero cells were infected in a 6-well plate with DENV-2 or CHIKV at MOI of 0.25 in both mono-infection and co-infection assays and then evaluated at 48 h. The cells were harvested from each plate, then double stained assay and flow cytometry analysis were performed to confirm cells infected with DENV anti-NS5 (X) and infected cells with CHIKV anti-E2 (Y). (TIFF 917 kb) [file 13071_2018_2942_MOESM1_ESM.tiff]
